# Fecal microbiota dynamics during disease activity and remission in newly diagnosed and established ulcerative colitis

**DOI:** 10.1038/s41598-021-87973-7

**Published:** 2021-04-21

**Authors:** Lena Öhman, Anders Lasson, Anna Strömbeck, Stefan Isaksson, Marcus Hesselmar, Magnus Simrén, Hans Strid, Maria K. Magnusson

**Affiliations:** 1 Department of Microbiology and Immunology, Institute for Biomedicine, Sahlgrenska Academy, University of Gothenburg, Box 435, 405 30 Gothenburg, Sweden; 2 Department of Internal Medicine and Clinical Nutrition, Institute for Medicine, Sahlgrenska Academy, University of Gothenburg, Gothenburg, Sweden; 3 Department of Internal Medicine, Södra Älvsborg Hospital, Borås, Sweden; 4 Center for Functional Gastrointestinal and Motility Disorders, University of North Carolina at Chapel Hill, Chapel Hill, NC USA

**Keywords:** Microbiome, Dysbiosis, Inflammatory bowel disease

## Abstract

Patients with ulcerative colitis (UC) have an altered gut microbiota composition, but the microbial relationship to disease activity needs to be further elucidated. Therefore, temporal dynamics of the fecal microbial community during remission and flare was determined. Fecal samples were collected at 2–6 time-points from UC patients during established disease (cohort EST) and at diagnosis (cohort NEW). Sampling range for cohort EST was 3–10 months and for cohort NEW 36 months. Relapses were monitored for an additional three years for cohort EST. Microbial composition was assessed by Genetic Analysis GA-map Dysbiosis Test, targeting ≥ 300 bacteria. Eighteen patients in cohort EST (8 with maintained remission and 10 experiencing a flare), provided 71 fecal samples. In cohort NEW, 13 patients provided 49 fecal samples. The microbial composition showed no clustering related to disease activity in any cohort. Microbial dissimilarity was higher between than within patients for both cohorts, irrespective of presence of a flare. Microbial stability within patients was constant over time with no major shift in overall composition nor modification in the abundance of any specific species. Microbial composition was not affected by intensified medical treatment or linked to future disease course. Thus in UC, the gut microbiota is highly stable irrespective of disease stage, disease activity or treatment escalation. This suggests that prolonged dietary interventions or repeated fecal transplantations are needed to be able to induce permanent alterations of the gut microbiota.

## Introduction

Ulcerative colitis (UC) is a chronic inflammatory disease of the colon with a disease course characterized by periods of active disease with flares of abdominal pain, diarrhea and hematochezia followed by periods of remission^1^. Multiple cross-sectional studies have demonstrated that patients with inflammatory bowel disease (IBD), comprising UC and Crohn’s disease (CD), have an altered gut microbiota composition as compared to healthy individuals, with lower microbial richness and diversity as compared to healthy individuals, reviewed in^2,3^. These differences emphasize the potential role for the gut microbiota for development and/or progression of IBD.

Longitudinal profiling studies demonstrate that the intra-individual stability of the microbial composition over time is high in both healthy individuals and IBD patients^4,5^, although with a higher degree of fluctuation in CD patients^2,6,7^. However, few studies have explored gut microbiota dynamics in relationship to disease activity over time. Studies that prospectively monitor gut microbiota in IBD before and during a flare of the disease report no evidence for overall changes in microbiota composition linked to the flare^6,8,9^, even though some patient-specific taxonomic shifts have been detected^8^. Recent studies further demonstrate that the fecal microbiota composition of CD patients is subject to little intra-individual variation over time and is not influenced by disease activity, neither in the short- nor long-term perspective^9,10^. However, the relationship between gut microbiota dynamics and the disease activity during different phases of the disease in UC still needs to be explored. Also, multiple sampling times spanning before, during and after a flare are warranted. Therefore, in this longitudinal study we determined the temporal dynamics of fecal microbiota composition during remission and flare in UC patients with either established disease or at diagnosis.

## Results

### Study populations

**Figure 1:**
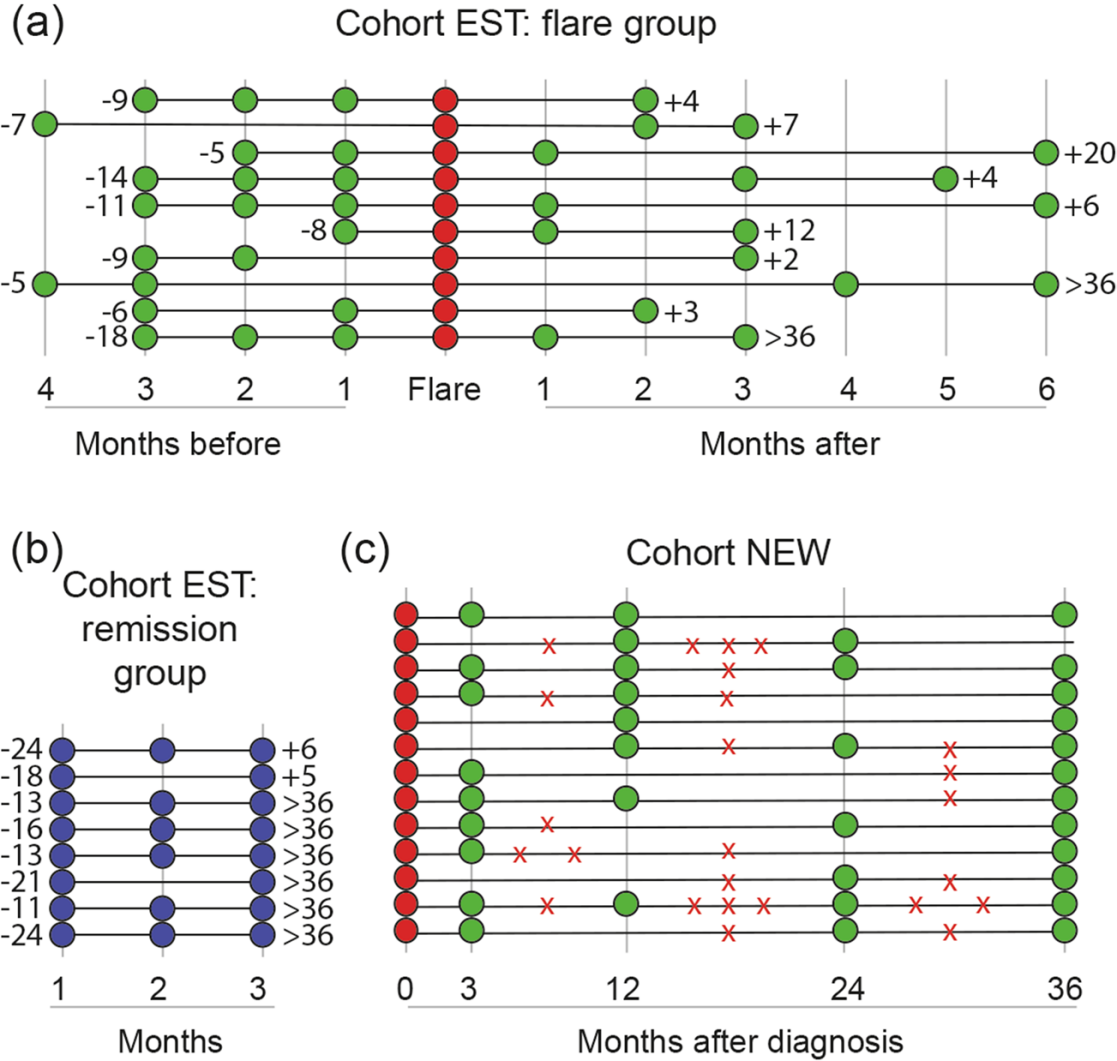
Time intervals for fecal sampling and time indication for flares for the study cohorts. Fecal samples were obtained at time-points indicated by the circles, each row represents one patient. Sampling for patients with established UC (cohort EST) are divided into **(A)** flare group and **(B)** remission group. Numbers to the left indicate months to previous flare and numbers to the right indicate months to the next flare. **(C)** Sampling time-points for patients with newly diagnosed UC (cohort NEW). The symbol X indicates the time period when the patient experienced a flare. Note that the X does not indicate an exact time-point, the flare occurred any time during the specific time interval.

For cohort EST, 39 patients were included to the initial study and 17 experienced a flare. Of these 17, 10 patients fulfilled the inclusion criteria for this study. In addition, the 8 first included patients fulfilling the criteria for maintained remission were selected. For patient demographics see Table 1, time intervals for fecal sampling and relation to flares before and after sampling are shown in Fig. 1A,B. Together the patients provided 71 fecal samples. At inclusion, when all patients were in remission, 17 of the patients had an endoscopic mayo score of 0 while one patient had an endoscopic mayo score of 1 (belonging to the flare group). All patients were on stable 5ASA treatment and one patient was on concomitant treatment with azathioprine since 9 months. During remission, medicines were kept stable. Presence of a flare was determined by sigmoidoscopy (n = 5) or by an increase in symptoms and calprotectin levels > 300 µg/g (n = 5). During a flare, treatment included standardized 5-ASA dose escalation without (N = 7) or with oral corticosteroids (N = 3). During follow-up, three years after the last study sample, median flare numbers were 0 (range 0–3) and 2 (0–4), for remission and flare group, respectively (p = 0.01).

**Table 1:** Patient demographics at inclusion for patients during established disease (cohort EST) and at diagnosis (cohort NEW).

	Cohort EST		p-value	Cohort NEW (n = 13)
	Remission group (n = 8)	Flare group (n = 10)		
Age (median, range)	40 (31–60)	36 (20–50)	0.32	34 (22–55)
Gender (female)	4	6	0.67	7
Disease extent (E1/E2/E3^a^)	0/3/5	0/6/4	0.34	2/2/9
Disease duration, years (median, range)	3 (1–13)	5 (1–16)	0.36	NA
Smoking (no/yes)	7/1	9/1	0.87	12/1
Fecal calprotectin, µg/g (median, IQR)	40 (20–80)	50 (30–90)	0.59	650 (380–1600)
Months since previous flare, median (range)	17 (11–24)	9 (5–18)	0.006	NA
**Maintenance treatment**			0.36	NA
5ASA	8	9		
5ASA/AZA	0	1		

*NA* not applicable, *5ASA* 5-aminosalicylic acid, *AZA* azathioprine.

^a^E1, proctitis; E2, left-sided colitis; E3, extensive colitis.

For cohort NEW, 100 patients were included to the initial study but only 13 patients had enough quantity of stool sample available for analysis from the visit at diagnosis and could enter this study (Table 1), resulting in 49 fecal samples. Time intervals for fecal sampling and flares during the 36 months sampling time are shown in Fig. 1C. Treatment of the flare at diagnosis included 5-ASA without (N = 3) or with corticosteroids (N = 10, 3 topical, 7 oral). After inducing remission from the initial flare, two patients maintained in remission, four experienced 1 flare and seven experienced ≥ 2 flares the coming 3 years.

### Fecal microbiota dynamics over time in UC

**Figure 2:**
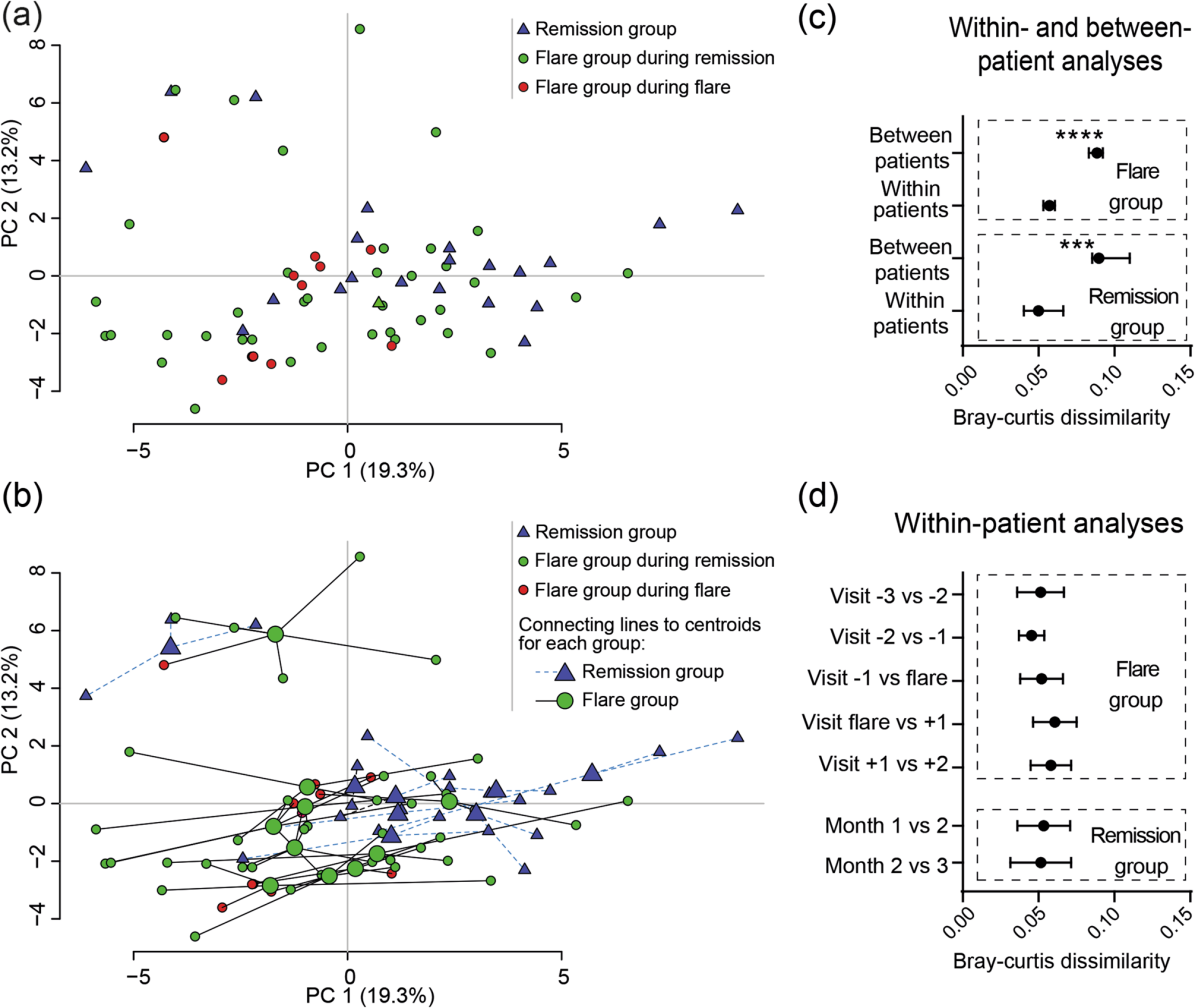
Fecal microbiota dynamics during remission and active flare in patients with established UC. Fecal samples were obtained from patients with established UC (cohort EST) and analysed by the GA-map Dysbiosis Test (remission N = 8, flare N = 10, total number of samples N = 71). The flare group include samples from visit − 3, − 2, − 1, flare, + 1 and + 2 where samples − 3 to − 1 were obtained 1–4 months before the flare and samples + 1 to + 2 were obtained 1–6 months after the flare. The remission group samples were obtained at two to three months in a row. **(a,b)** Principal component analysis showing remission group (blue triangles), flare group during remission (green circles) and flare group during active flare (red circles). In **(b)** samples originating from individual patients are linked to their centroids by dotted lines (remission group) or solid lines (flare group). **(c)** Within- and between-patient and **(d)** within-patient microbial dissimilarities for flare and remission group. The dissimilarities were analyzed by Bray–Curtis dissimilarity index. Data in **(c,d)** are shown as median (IQR), ***p < 0.001, ****p < 0.0001.

Temporal dynamics in microbial composition was first evaluated for cohort EST. Principal component analysis (PCA) of all samples, coded for remission group and flare group during either remission or flare showed no clustering for any of the groups (Fig. 2A). Linking of intra-individual samples to their centroid in the PCA revealed that samples from the same individual tended to localize close to one another (Fig. 2B). This was confirmed by assessing Bray–Curtis dissimilarities, which quantify the compositional dissimilarity between different samples based on the microbial probe intensities. Results showed that the dissimilarity was higher between patients than within patients, both for the remission and flare group (Fig. 2C). Next, we analyzed temporal microbial dissimilarities within patients for the remission and flare groups, which showed constant stability over time with no alterations before, during or after a flare (Fig. 2D).

**Figure 3:**
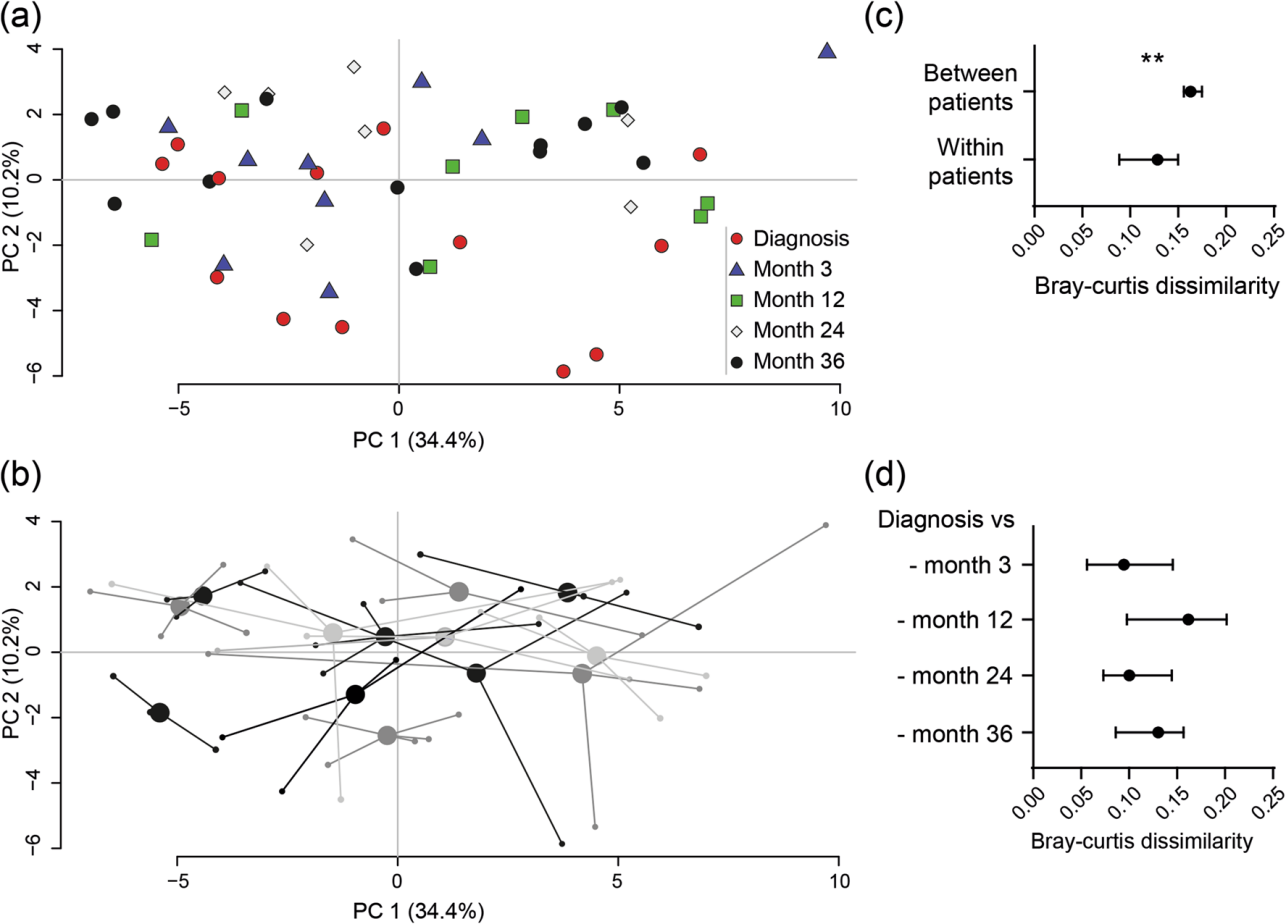
Fecal microbiota dissimilarity dynamics in patients with newly diagnosed UC. Fecal samples were obtained from patients with newly diagnosed UC (cohort NEW) and analysed by the GA-map Dysbiosis Test (N = 13, total number of samples N = 49). Samples were obtained at diagnosis and at month 3, 12, 24 and 36. Principal component analyses with **(a)** the sampling time indicated by color/shape of the dots and **(b)** samples originating from individual patients linked to their centroids. **(c)** within- and between-patient and **(d)** within-patient microbial dissimilarities analyzed by Bray–Curtis dissimilarity index. Data are shown as median (IQR), **p < 0.01.

Then, we examined alterations in microbial composition for cohort NEW. PCA analysis showed no specific clustering linked to active disease at diagnosis (Fig. 3A) and samples from the same individual tended to localize close to one another (Fig. 3B). Again dissimilarities were higher between than within patients (Fig. 3C) and temporal microbial dissimilarities were stable over time when compared to the time for diagnosis (Fig. 3D).

**Figure 4:**
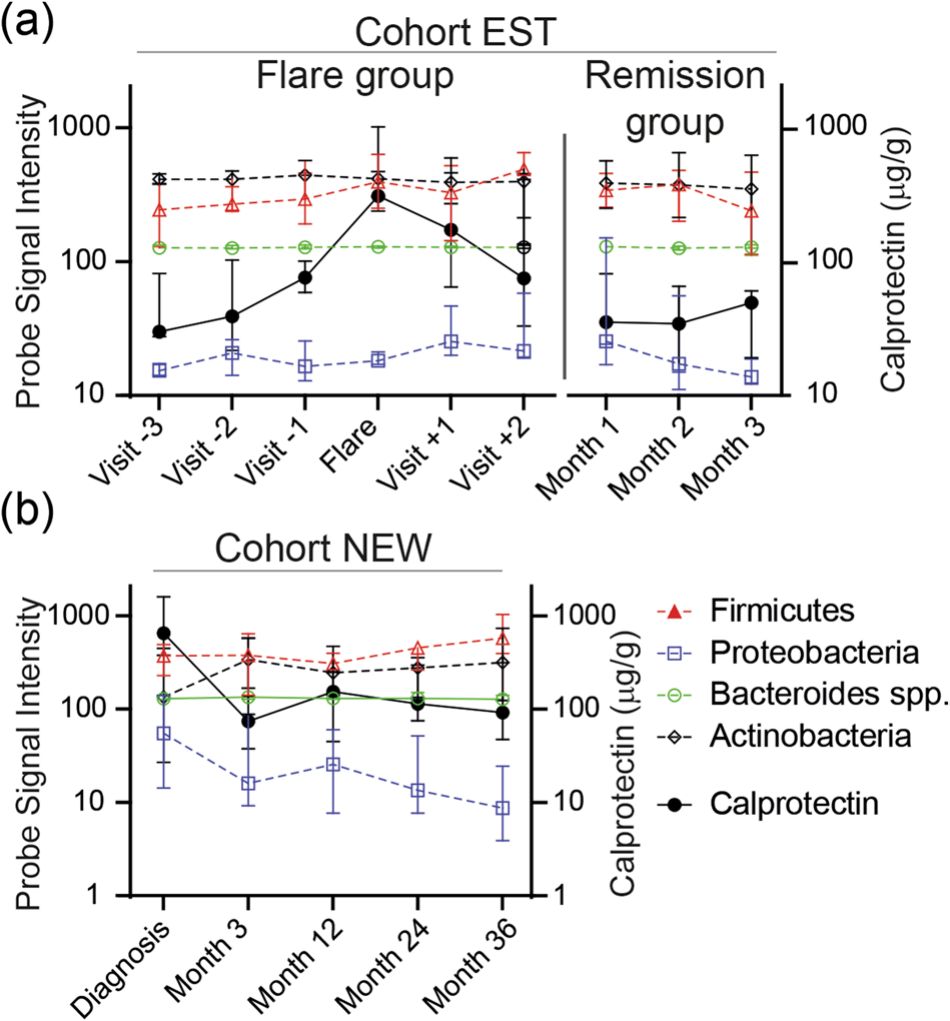
Bacterial probe intensity for the four major phyla in relation to levels of calprotectin in fecal samples during remission and active flare in patients with established and newly diagnosed UC. Fecal samples were analysed by the GA-map Dysbiosis Test and for levels of calprotectin by ELISA. **(a)** Patients with established disease (cohort EST, remission N = 8, flare N = 10, total number of samples N = 71). The flare group include 1–3 samples before, 1 during and 1–2 after the flare and the remission group includes samples obtained two to three months in a row. **(b)** Patients with newly diagnosed UC (cohort NEW, N = 13, total number of samples N = 49). Probe signal intensity for the bacteria is indicated on the left y-axis and calprotectin concentration on the right y-axis. Lines linking bacteria are dotted and symbols are open, lines linking calprotectin are solid and symbols are filled. The Probe Signal Intensity shows absolute values and can be compared between different samples, however a high Probe Signal Intensity for one bacterial species does not imply higher presence when compared to a low signal of a different bacterial species. Data are shown as median (interquartile range).

**Figure 5:**
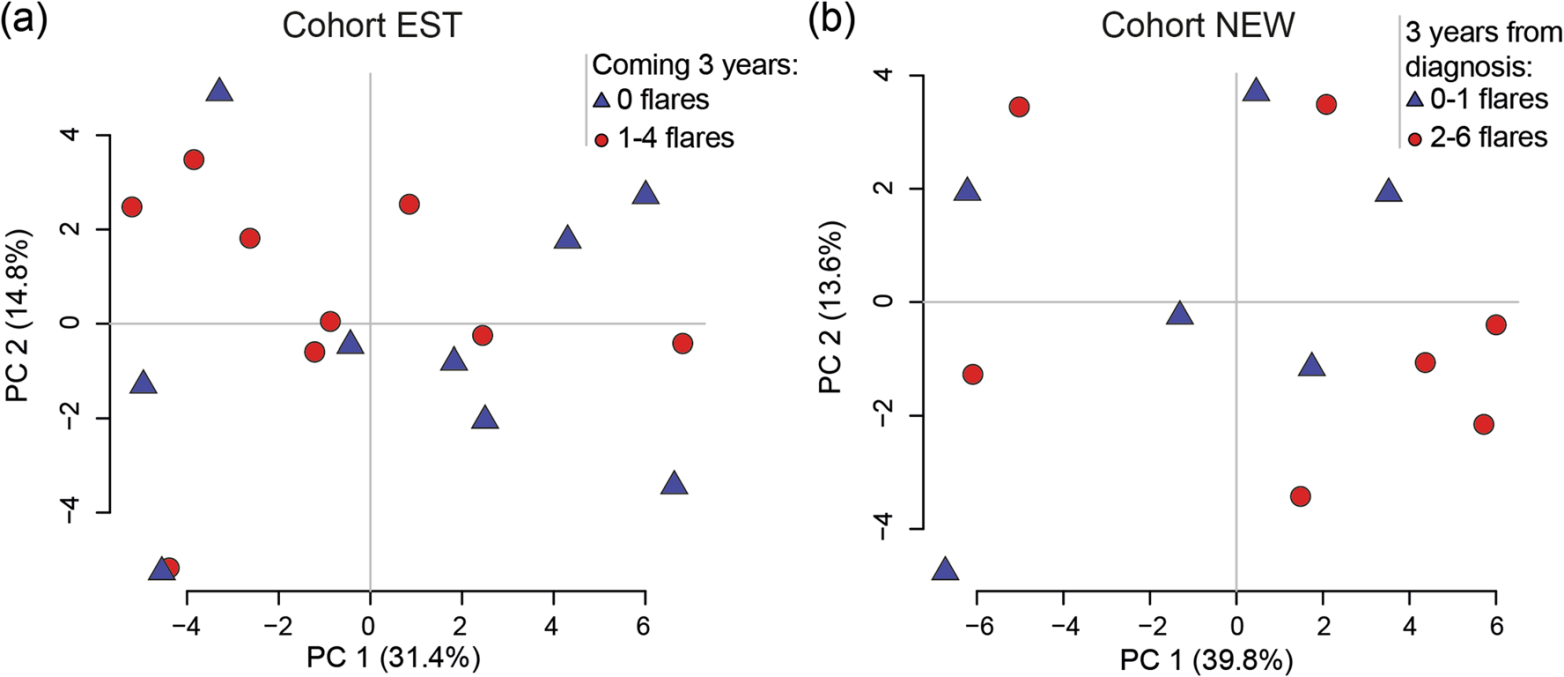
Fecal microbiota and disease outcome the coming 3 years. Fecal samples were obtained from patients with UC and analysed by the GA-map Dysbiosis Test. The microbial compositions are shown by principal component analyses. **(a)** For patients with established disease (cohort EST), results for the last sample obtained is shown in relation to disease outcome after 3 years grouped into maintained remission (N = 9) and 1–4 flares (N = 9). **(b)** For patients at diagnosis (cohort NEW), results for the first sample obtained is shown in relation to disease outcome after 3 years grouped into 0–1 flares (N = 6) and 2–6 flares (N = 7).

Despite a change in fecal calprotectin levels at the flare for cohort EST (visit -1 *vs.* flare; 80 µg/g (60–190) *vs.* 310 µg/g (250–1020), p < 0.0001) and at diagnosis for cohort NEW (inclusion *vs.* month 3; 650 µg/g (380–1600) *vs.* 70 µg/g (40–570), p = 0.03) there were no significant changes in probe signal intensity over time for the four major phyla, Firmicutes, Bacteroides spp., Proteobacteria or Actinobacteria, in any of the cohorts (Fig. 4A,B), or for any of the other 50 probes detecting bacteria on different taxonomic levels (data not shown). No effect related to treatment escalation could be detected for the flare group of cohort EST between Flare and Visit + 1 (Fig. 4A, left) or for initiation of treatment in cohort NEW comparing diagnosis and month 3 (Fig. 4B). Finally, there was no link between the microbiota composition and manifestations of flares the coming 3 years in any of the patient cohorts (Fig. 5).

## Discussion

In this longitudinal study of UC patients, we have demonstrated that the microbiota composition is constant over time with no alterations before, during, or after a flare of the disease or during remission, both at the time of diagnosis and in established UC. Our results further show that the microbial dissimilarity is higher between than within patients, irrespective of disease activity or time since diagnosis, supporting the notion of the long-term stability of an individual’s gut microbiota.

During the last decade several studies have reported differences in fecal microbial composition between active and inactive IBD^6,11–13^. However, most previous studies have had a cross-sectional design comparing fecal microbiota from patients with active disease and patients in remission. Our results clearly show that the inter-individual differences in fecal microbiota composition are larger than the intra-individual differences over time, confirming previous reports on the matter^8,9^. Thus, differences in fecal microbiota composition related to disease activity demonstrated using a cross sectional set-up most likely reflect individual differences in microbiota composition rather than disease activity.

More recently, data from long-term investigations have suggested that gut microbiota composition within an individual is relatively constant over time, although microbiota of IBD patients has been proposed to fluctuate more than those of healthy subjects^6,8–10^. Potential instability of gut microbiota of IBD patients may be linked to inflammation and our approach was therefore to follow patients before, during and after flare of disease. Our study demonstrates a high microbial stability over time in UC, both at the time of diagnosis and with established disease, with no changes in the overall microbial composition related to disease activity. Moreover, the high stability of the microbial profile during the three years after time of diagnosis indicates that the deviation from a healthy microbiota was firmly established before or during early development of disease. Thus, by including several consecutive samples obtained before, during, and after a flare of the disease linked to thorough clinical follow-up we confirm the stability of the microbiota community both at the time of diagnosis and in established UC.

Similar to the lack of influence by disease activity, increased dose of 5ASA or addition of corticosteroids did not affect the microbial community in the present study, neither when being introduced to patients with newly diagnosed disease, nor when being intensified in patients with established disease. Comparable results have recently been reported for both CD and UC with no impact of medication on microbial stability^9,14^. Nevertheless, microbial diversity was improved in pediatric IBD patients responding to anti-TNF therapy^15^. The inconsistent reports of potential impact of medical regimens on gut microbiota raises the question whether the different outcomes is related to the type of drug or that gut microbiota of adolescent patients are more volatile than that of adult patients. It might also be considered if the stability of the microbiota profile in adult IBD patients may be a potential explanation for fecal transplantation only partly meeting the high expectations as a therapeutic option for IBD patients^16,17^. Furthermore, the usefulness of autologous fecal transplantation, i.e. one’s own microbiota, for treatment in IBD patients could also be disputed^18^. Concerning diets and the microbiota, UC patients on vegetarian or gluten free diets show altered gut microbiota profiles in comparison to omnivores but without any clinical benefits^19^. For diet interventions, a study employing a low fermentable oligosaccharides, disaccharides, monosaccharides and polyols (FODMAP) diet for 4 weeks in IBD patients reported higher relief of gut symptoms *vs.* normal diet and showed lower abundance of *Bifidobacterium adolescentis*, *Bifidobacterium longum* and *Feacalibacterium prausnitzii* but no alterations in phyla distribution or α- and β-diversity^20^. A 4-week diet of low fat, high fiber was evaluated in UC patients in comparison to an improved standard American diet and resulted in decreased markers of inflammation and reduced dysbiosis^21^. However, long term persistence of the altered microbiota is still unknown and considering the high stability of the microbiota such studies are warranted. Altogether this further emphasizes the need of longitudinal studies evaluating the dynamics of gut microbiota related to different treatment regimens including medical therapies, diets and fecal transplantation.

The main strength of our study is the longitudinal study design, which allows for assessment of associations between microbiota composition and disease activity within patients over time; this is particularly important considering the inter-individual differences in microbiota composition and medication use. Compared to previous studies with similar research focus^8,9^, we have included more samples per individual, for the established phase encompassing time-points both before and after the flare to evaluate possible changes preceding or following a flare, and for the newly diagnosed encompassing both short- and long-term sampling. We also used a different mode of analyzing the microbiota, as the GA-map technology is based on a pre-selected set of bacteria known to discriminate IBD patients from healthy subjects. The use of the GA-map Dysbiosis Test has limitations since it only determines defined bacterial sequences, ruling out the possibility to find new strains or to deliver full taxonomic details and α and β diversity values. On the other hand, strengths of the method include the generation of absolute values instead of relative abundance, enabling direct comparisons between samples, and the ease of data handling as compared to deep or shotgun sequencing.

A limitation of our study is that the limited number of patients, however, the standardized treatment regimens, the large number of samples per individual and the use of two different cohorts strengthen the findings of the study. Another limitation is that we have not ruled out the presence of an infectious agent, such as Clostridium difficile, as a cause for the flares in the established cohort. However, symptoms were consistent with UC exacerbation and the patients responded well to an increased dose of 5-ASA with or without corticosteroids. Also, no conclusions concerning thiopurines and microbiota could be drawn for this study since only one patient was on thiopurines, but a previous study has reported decreased alpha-diversity in patients treated with thiopurines^8^.

In summary, we have shown that the gut microbiota in UC is highly stable irrespective of stage of the disease, disease activity or treatment escalation. This suggests that previous studies showing differences in microbiota composition between patients in remission and patients experiencing a flare reflect individual differences, rather than disease activity. It also suggests that nutritional modulation of the gut microbiota as a strategy to control the disease requires long-term commitment.

## Methods

### Study population

This study encompassed two cohorts of UC patients recruited from five gastroenterology units in Western Sweden; cohort EST comprised patients with established disease being in remission at inclusion, whereas cohort NEW comprised patients included at the time for diagnosis.

Cohort EST was initially recruited from the control group of a 5-ASA intervention study^22^. Patients were > 18 years old, on standardized maintenance treatment with oral 5-ASA. Exclusion criteria were ongoing anti-TNF, corticosteroid or non-steroidal anti-inflammatory drug treatment, pregnancy or prior colon resection. At inclusion, all patients were in remission defined as a Mayo score ≤ 2, with no single variable > 1, confirmed by a flexible sigmoidoscopy. Patients were asked to provide stool samples by regular mail every month during 1.5 years, but the compliance of sending samples differed from patient to patient. A relapse was defined by colonoscopy or by an increase in symptoms, consistent with UC, together with a calprotectin level > 300 µg/g. For the present study, selection criteria for patients with a flare included clinically diagnosed relapse with 1–3 stool samples before the flare (1–4 months prior), one stool sample at the flare and 1–2 stool samples after the flare (1–6 months post). Selection criteria for patients without a relapse were 2–3 stool samples during consecutive months with no flares the previous six months. The remission group was chosen to be of similar size as the relapse group and patients were included by date order (from inclusion start). None of the patients had been taking antibiotics the last month prior to sample collection or did so during the sampling time. After the last stool sample, numbers of relapses were monitored for an additional three years.

Cohort NEW was included at the time for diagnosis and consisted of patients with new onset of UC^23^. Inclusion criteria were newly diagnosed UC, age 18–75 years without medical treatment for IBD. Exclusion criteria were other severe diseases such as heart, lung, or neurological disease, and active malignancies, as well as antibiotic use during the month before inclusion. The extent of the disease and the endoscopic disease activity were established with colonoscopy or sigmoidoscopy. Stool samples were sent by regular mail at the time of the diagnosis and at month 3, 12, 24 and 36. Numbers of relapses were monitored until month 36.

Stool samples from both cohorts arrived within 24 h from sampling to the clinic and were immediately frozen at – 20 ˚C. Within one week from sampling the fecal calprotectin was determined by ELISA according to the manufacturer’s instructions (Bühlmann Laboratories AG, Basel, Switzerland). Detection range was 10–600 µg/g and samples above the higher threshold were diluted and re-run.

Sample storage experiments at room temperature have shown stability up to 3 days for calprotectin^24^ and 5 days for the GA-map Dysbiosis Test^25^.

### Microbiota analysis, data analyses and statistics

Targeted microbiota analysis of 1 g of fecal samples was performed using the commercially available GA-map Dysbiosis Test (Genetic Analysis AS, Oslo, Norway). The GA-map Dysbiosis Test consists of 54 DNA probes targeting ≥ 300 bacteria on different taxonomic levels assessed as Probe Signal Intensity^25^. Probes are listed in Supplementary Table S1.

Statistical and data analyses were performed in IBM SPSS 25 and R Studio 1.1.456 (R 3.6.1), respectively. Principal component analyses were performed using the pca3d package in R. Bray–Curtis dissimilarity was used in the R vegan package to investigate differences within and between study subjects. Within-patient analyses over time show dissimilarities between one sample to the next for the same patient (established disease) or diagnosis sample to each of the following samples for the same patient (newly diagnosed). Within- vs. between-patient analyses show mean of all dissimilarities between samples for a unique patient (= within) vs. mean of all dissimilarities for a unique patient to all non-related samples (= between). Lower numbers infer higher similarity between samples. For *post-hoc* analysis, Mann–Whitney U test or Kruskal–Wallis test followed by Dunn’s multiple comparisons test were used. For demographic patient data, Mann–Whitney U and chi-squared tests were used. Data are shown as median (interquartile range, IQR) unless otherwise stated.

### Ethical considerations

All subjects provided their verbal and written informed consent before participation according to the declaration of Helsinki. Approval was obtained by the Regional Ethical Review Board in Gothenburg prior to start of the studies (Dnr 403-12 and 154-09).

## Supplementary Information

Supplementary Table S1.
