# Supplementary material for: Fecal microbiota dynamics during disease activity and remission in newly diagnosed and established ulcerative colitis
Source: Sci Rep. 2021 Apr 21;11:8641. doi: 10.1038/s41598-021-87973-7 (PMC8060394; doi:10.1038/s41598-021-87973-7)
Supplement: Supplementary file 1 — Supplementary Table S1. [file 41598_2021_87973_MOESM1_ESM.docx]

**Fecal microbiota dynamics during disease activity and remission in newly diagnosed and established ulcerative colitis**

Lena Öhman^1,2^, Anders Lasson^3^, Anna Strömbeck^1^, Stefan Isaksson^1^, Marcus Hesselmar^2^, Magnus Simrén^2,4^, Hans Strid^3^ and Maria K Magnusson^1^.

*^1^University of Gothenburg, Inst. for Biomedicine, Sahlgrenska Academy, Dept. of Microbiology and Immunology, Gothenburg, Sweden, ^2^University of Gothenburg, Inst. for Medicine, Sahlgrenska Academy, Dept. of Internal Medicine and Clinical Nutrition, Gothenburg, Sweden and ^3^Dept. of Internal Medicine, Södra Älvsborg Hospital, Borås, Sweden, ^4^Center for Functional Gastrointestinal and Motility Disorders, University of North Carolina at Chapel Hill, Chapel Hill, North Carolina.*

**Supplementary Table S1.** List of the 54 probes included in **GA-map** Dysbiosis Test; bacteria names and taxonomy.

| **Phylum** | **Class** | **Genus/Species** |
| --- | --- | --- |
| Actinobacteria | Actinobacteria | Actinobacteria |
| Actinobacteria | Actinopbacteria | Actinomycetales |
| Actinobacteria | Corinobacteria | *Atopobium rimae* |
| Actinobacteria | Actinobacteria | *Bifidobacterium* spp. |
| Bacteroidetes | Alistipes | *Alistipes* |
| Bacteroidetes | Alistipes | *Alistipes onderdonkii* |
| Bacteroidetes | Bacteroides | *Bacteroides fragilis* |
| Bacteroidetes | Bacteroides | *Bacteroides pectinophilus* |
| Bacteroidetes | Bacteroides | *Bacteroide*s spp. |
| Bacteroidetes | Bacteroides | *Bacteroides* spp. & *Prevotell*a spp. |
| Bacteroidetes | Bacteroides | *Bacteroides stercoris* |
| Bacteroidetes | Bacteroides | *Bacteroides zoogleoformans* |
| Bacteroidetes | Parabacterioides | *Parabacteroides johnsonii* |
| Bacteroidetes | Parabacterioides | *Parabacteroides* spp. |
| Bacteroidetes | Prevotella | *Prevotella nigrescens* |
| Firmicutes | Negativicutes/Clostridia | Firmicutes |
| Firmicutes | Clostridia | *Anaerotruncus colihominis* |
| Firmicutes | Bacilli | Bacilli |
| Firmicutes | Bacilli | *Bacillus megaterium* |
| Firmicutes | Erysipelotrichia | *Catenibacterium mitsuokai* |
| Firmicutes | Clostridia | Clostridia |
| Firmicutes | Ruminiclostridium | *Clostridium methylpentosum* |
| Firmicutes | Clostridia | *Clostridium* sp. |
| Firmicutes | Erysipelotrichia | *Coprobacillus cateniformis* |
| Firmicutes | Clostridia | *Desulfitispora alkaliphila* |
| Firmicutes | Negativicutes | *Dialister invisus* |
| Firmicutes | Negativicutes | *Dialister invisus* & *Megasphaera micronuciformis* |
| Firmicutes | Clostridia | *Dorea* spp. |
| Firmicutes | Clostridia | *Eubacterium biforme* |
| Firmicutes | Clostridia | *Eubacterium hallii* |
| Firmicutes | Clostridia | *Eubacterium rectale* |
| Firmicutes | Clostridia | *Eubacterium siraeum* |
| Firmicutes | Clostridia | *Faecalibacterium prausnitzii* |
| Firmicutes | Clostridia | Lachnospiraceae |
| Firmicutes | Bacilli | *Lactobacillus ruminis* & *Pediococcus acidilactici* |
| Firmicutes | Bacilli | *Lactobacillus* spp. |
| Firmicutes | Bacilli | *Lactobacillus* spp. 2 |
| Firmicutes | Negativicutes | *Phascolarctobacterium* sp. |
| Firmicutes | Clostridia | Ruminococcus albus & R. bromii |
| Firmicutes | Clostridia | *Ruminococcus gnavus* |
| Firmicutes | Bacilli | *Streptococcus agalactiae* & *Eubacterium rectale* |
| Firmicutes | Bacilli | *Streptococcus salivarius* ssp. *thermophilus* & S. *sanguinis* |
| Firmicutes | Bacilli | *Streptococcus salivarius* ssp.*thermophilus* |
| Firmicutes | Bacilli | *Streptococcus* spp. |
| Firmicutes | Bacilli | *Streptococcus* spp. 2 |
| Firmicutes | Negativicutes | *Veillonella* spp. |
| Firmicutes/Tenericutes/Bacteroidetes species |  | Firmicutes (various) |
| Proteobacteria | Proteobacteria | Proteobacteria |
| Proteobacteria | Gammaproteobacteria | *Acinetobacter junii* |
| Proteobacteria | Gammaproteobacteria | Enterobacteriaceae |
| Proteobacteria | Gammaproteobacteria | *Pseudomonas* spp. |
| Proteobacteria | Gammaproteobacteria | *Shigella* spp. & *Escherichia* spp. |
| Tenericutes | Mollicutes | *Mycoplasma hominis* |
| Verrucomicrobia | Verrumicrobiae | *Akkermansia muciniphila* |
